# Integrated, rational molecular replacement

**DOI:** 10.1107/S2059798321001339

**Published:** 2021-02-05

**Authors:** Isabel Usón, Charles C. Ballard, Ronan M. Keegan, Randy J. Read

**Affiliations:** a Crystallographic Methods, Institute of Molecular Biology of Barcelona (IBMB-CSIC), Baldiri Reixach 15, 08028 Barcelona, Spain; b ICREA, Pg. Lluís Companys 23, 08010 Barcelona, Spain; c CCP4, Research Complex at Harwell, Rutherford Appleton Laboratory, Harwell Oxford, Didcot, OX11 0FA, United Kingdom; d Cambridge Institute for Medical Research, Department of Haematology, University of Cambridge, The Keith Peters Building, Hills Road, Cambridge, CB2 0XY, United Kingdom

**Keywords:** CCP4 Study Weekend, molecular replacement, integrated structural biology

## Abstract

Introducing the virtual special issue on the 2019 CCP4 Study Weekend on Integrated, rational molecular replacement at https://journals.iucr.org/special_issues/2020/CCP42019/.

In the past 50 years, molecular replacement (MR) has grown from being a way to shortcut the occasional determination of identical or closely related structures to being the route to the majority of protein crystal structures. The 2019 CCP4 Study Weekend on Molecular Replacement, held on 9–10 January 2019 in Nottingham, UK, looked briefly at its long history but focused on recent developments that have helped to push its reach to a much greater range of structures. The special issue at https://journals.iucr.org/special_issues/2020/CCP42019/ contains contributions from the majority of the speakers at the meeting.

After an overview of the history of molecular replacement by Eleanor Dodson, Airlie McCoy talked about the development of likelihood-based MR, and how the use of graph databases will allow even more sophisticated branched searches testing different structural hypotheses. The likelihood approach benefits from accounting for the effects of errors, which must be estimated in advance for structural models. Björn Wallner discussed methods to accurately estimate the local errors in computational models, which can be used to weight the relative contributions of different parts of these models. Kaushik Hatti focused on global errors, discussing how these can be estimated from a combination of factors including sequence similarity scores, model size, and model quality scores. Randy Read examined the connection between likelihood scores and measures of information gain, showing that information gain can be used to choose the data that will contribute usefully to MR calculations.

The application of MR can be hindered by problems with the crystals, so it helps to diagnose and understand crystal pathologies. Regine Herbst-Irmer discussed the case of non-merohedral twinning and how this can be dealt with in a variety of circumstances. Iracema Caballero examined issues raised by translational non-crystallographic symmetry: how it can be detected, characterized, and accommodated in structure solution. Another surprisingly common problem is for crystals to grow from the wrong component from the protein purification; Adam Simpkin discussed an approach using a database of ensembles of folds to solve such structures, by detecting which contaminant actually crystallized.

Some of the most exciting advances in the past few years have come from the development of methods to solve structures starting from much smaller fragments, including generic fragments such as helices. Several talks focused on new developments on this front that build on better understanding of structural patterns and integration with other computational methods. Dan Rigden showed that success in using *ab initio* computational models can be improved by testing a variety of pruned models. Ana Medina discussed the detection and preparation of generic compact folds and their use for fragment-based MR. Claudia Millán addressed a complementary problem: how to integrate a number of partial fragment-based solutions to build up a more complete picture. Coiled-coils represent a particularly difficult case for MR, and Owen Davies described new improvements in how they can be modelled. Electron diffraction is emerging as a promising alternative to X-ray diffraction for microcrystals, but it depends heavily on MR because conventional experimental phasing methods do not apply. Jennifer Miao discussed some of the issues with micro-electron diffraction, and demonstrated that MR can succeed with fragments generated from distant homologues because local structure is preserved better than global structure.

Even if generic fragments or compact folds can be placed correctly, expanding an incomplete starting model to a good final structure can be extremely difficult. Rafael Borges described a new algorithm to test a large variety of hypotheses for how the protein sequence can be assigned to the fragments, significantly extending model completeness. Grzegorz Chojnowski discussed new methods to detect and exploit local structural similarity between the target structure and distant homologues when rebuilding structures solved by MR. Carmelo Giacovazzo presented results from a new MR pipeline, which follows the MR placement with density modification and automated rebuilding.

The meeting ended with case studies describing the approaches used in some particularly difficult structure solutions. Paula Salgado outlined the path to the solution of a type IV pilin, pilA1. This started with attempts to solve the structure using models and ensembles derived from homologues, an approach that frequently succeeds even if not in this case; the structure eventually yielded to *ab initio* fragment-based phasing, aided by the insight that pilins typically possess a long helix. Montserrat Fàbrega-Ferrer discussed two ways in which low-resolution cryo-EM reconstructions can be used to solve a crystal structure, either by using the cryo-EM map as a model directly or (the approach used in practice in their work) using a partial atomic model built into that map.

One talk (not represented by a paper in this issue) foreshadowed a major recent development with enormous implications for the future of MR in protein crystallography. Andrew Senior presented striking *ab initio* modelling results achieved by deep learning algorithms in *AlphaFold*, which had been tested in the 13th edition of the Critical Assessment of Structure Prediction (CASP) experiment. In CASP14 late last year, its successor *AlphaFold*2 achieved even more astonishing results in predicting the structures of a wide variety of targets, in many cases achieving the levels of accuracy needed for successful MR.

We would like to thank the speakers for their excellent contributions to both the meeting and this collection of papers, and the CCP4 staff who did a great job of looking after the organizational details (Karen McIntyre, Helen Walker, Esme Williams and India Reeves) and audiovisual facilities (Stuart Eyres and Laura Bennett).

